# Case Report: CytoSorb hemoadsorption as an adjunctive therapy in critically ill pregnant women with COVID-19 and acute kidney injury—a case series

**DOI:** 10.3389/fmed.2025.1558768

**Published:** 2025-11-26

**Authors:** Minoo Heidari Almasi, Mir Mohammad Miri, Kiana Entezarmahdi, Tahereh Sabaghian, Soheila Sadeghi, Amirahmad Nassiri

**Affiliations:** 1 Imam Hossein Hospital Clinical Research Development Unit, Shahid Beheshti University of Medical Sciences, Tehran, Iran; 2 Anesthesiology and Critical Care Department, School of Medicine, Emam Hosein Hospital, Shahid Beheshti University of Medical Sciences, Tehran, Iran; 3 Department of ICU, Jam General Hospital, Tehran, Iran; 4 Department of Internal Medicine, Imam Hossein Hospital, Shahid Beheshti University of Medical Science, Tehran, Iran; 5 Department of Internal Medicine, School of Medicine, Imam Hossein Hospital, Shahid Beheshti University of Medical Sciences, Tehran, Iran; 6 Division of Nephrology, Department of Internal Medicine, Shahid Beheshti University of Medical Sciences, Tehran, Iran

**Keywords:** CytoSorb, hemoadsorption, CRRT, ICU, AKI, COVID-19, pregnancy

## Abstract

**Purpose:**

Critically ill pregnant women with COVID-19 are at high risk for acute kidney injury (AKI) and a hyperinflammatory state, contributing to multiorgan failure. This case series describes the adjunctive use of CytoSorb hemoadsorption therapy in this population.

**Methods:**

We present four critically ill pregnant women with COVID-19 pneumonia, AKI, and clinical signs of hyperinflammation. All patients were admitted to the intensive care unit (ICU) and received CytoSorb therapy, integrated with continuous renal replacement therapy (CRRT) or used as a standalone treatment. We analyzed clinical parameters (SOFA score, vasopressor requirement, PaO₂/FiO₂ ratio) and inflammatory biomarkers (C-reactive protein, ferritin) before and after CytoSorb initiation.

**Results:**

Following CytoSorb therapy, all four patients showed clinical improvement, evidenced by a reduction in SOFA scores, decreased vasopressor needs, and improved oxygenation. A concomitant decrease in inflammatory markers was also observed. All four patients survived and were discharged from the hospital in stable condition after an average ICU stay.

**Conclusion:**

In this small case series, adjunctive CytoSorb hemoadsorption was associated with rapid clinical stabilization and recovery in critically ill pregnant women with COVID-19, AKI, and hyperinflammation. These promising results warrant further investigation in controlled studies to confirm the efficacy and safety of this approach.

## Introduction

The severity of COVID-19 ranges from mild respiratory involvement to multiorgan failure, such as AKI. A scoping review and meta-analysis confirmed that AKI is a frequent and serious complication of COVID-19, associated with a significantly increased risk of mortality (1). Pregnancy itself poses an additional, higher risk for disease progression due to physiological and immunological alterations. Previous studies have shown that pregnancy increases the risk of AKI by 51%, regardless of comorbidities and age (2, 3). Therefore, the convergence of COVID-19-associated sepsis and the pro-thrombotic, pro-inflammatory state of pregnancy creates a high-risk scenario requiring urgent evaluation and management. It is also crucial to differentiate COVID-19 from other pregnancy-related complications, such as preeclampsia.

Research on COVID-19 in pregnancy remains scarce due to its incidence; however, a few studies have explored the role and safety of CRRT in this sensitive population (4, 5). Pregnant women with elevated inflammatory markers and advanced disease may benefit from cytokine and endotoxin clearance using medium or high cutoff membranes during CRRT (6).

While the safety of CRRT in pregnancy has been explored (4, 5), data on adjunctive blood purification techniques, such as cytokine hemoadsorption, in this vulnerable population are exceedingly scarce. CytoSorb, a hemoadsorption device, has been proposed to modulate the cytokine storm in severe COVID-19. Here, we report a case series of four critically ill pregnant women with COVID-19-associated AKI and hyperinflammation, who were managed with adjunctive CytoSorb hemoadsorption therapy integrated into their continuous renal replacement therapy (CRRT) circuit.

## Methods and case presentation

This retrospective case series includes four pregnant women with positive real-time polymerase chain reaction (RT-PCR) results for COVID-19, who were admitted to the ICU from July 2020 to May 2021. We included pregnant patients with severe symptoms, AKI, and hyperinflammation who were treated with CytoSorb. Critically ill patients were defined according to WHO criteria, including ARDS, sepsis and septic shock, thromboembolism, and/or multiorgan failure, including AKI (7).

Following standard protocols, initial treatment included hemodynamic management, antivirals (e.g., Remdesivir), and corticosteroids (e.g., dexamethasone 4 mg twice daily). Baseline characteristics, including symptoms and laboratory results, are presented in Table 1. Upon the onset of AKI, accompanied by a hyperinflammatory state and hemodynamic instability requiring escalating vasopressor support, adjunctive CytoSorb therapy was initiated. It was integrated into the continuous renal replacement therapy (CRRT) circuit, which was performed using the Diapact system (B. Braun, Melsungen, Germany) in continuous veno-venous hemofiltration (CVVH) mode. The CytoSorb adsorber was placed in the circuit before the dialyzer, and a heparin-based anticoagulation protocol was used.

**Table 1:** Clinical characteristics of admitted patients.

Characteristic	Case 1	Case 2	Case 3	Case 4
Demographics				
Age, years	31	32	29	40
BMI, kg/m^2^	29.6	33.2	25.4	29.2
Obstetrical history (gravid, para, live, abortion)	G2 P1 L1	G5 P3 L1 A1	G4 P1 L1 A2	G2 A1
Gestational week at admission (Weeks + Days)	23 + 2	28 + 2	24 + 5	34 + 3
Clinical course				
Chief complaint	Cough, Dyspnea, Chilling	Cough, Dyspnea, Fatigue	Cough, Dyspnea, Chilling, Diarrhea	Cough
Number of hospital days (ICU)	9	27	20	15
Complications	Bilateral Pneumonia, AKI, sepsis	Bilateral Pneumonia, ARDS, AKI	Bilateral Pneumonia, AKI, sepsis	Bilateral Pneumonia, ARDS, sepsis
Duration of mechanical ventilation, days	-	12	-	11
CRRT mode	CVVH	CVVH	CVVH	CVVH
CRRT time, hours	24	42	155	24
APACHE II score	7	7	8	9

Laboratory results	Before / During / After CRRT	Before / During / After CRRT	Before / During / After CRRT	Before / During / After CRRT
WBC (×10^3^/μL)	10.8 / 11.9 / 15.12	10.7 / 12.5 / 17.5	8.2 / 16.0 / 13.6	14.3 / 15.8 / 13.8
Lymphocyte (×10^3^/μL)	8.8 / 9.4 / 12.8	16.0 / 14.7 / 17.3	14.1 / 5.5 / 10.4	15.4 / 14.2 / 14.8
Platelet (×10^3^/μL)	276 / 329 / 360	224 / 293 / 235	294 / 178 / 193	358 / 281 / 253
Urea (mg/dL)	24 / - / 11.2	11 / - / 11	12 / - / 34.5	24 / - / 22
Creatinine (mg/dL)	0.7 / - / 0.7	0.6 / - / 0.5	0.7 / - / 0.6	0.6 / - / 0.5
d-dimer (ng/mL)	650 / 460 / 2,891	1,254 / 2023 / 3,256	783 / 1,656 / 2,131	565 / 798 / 965
LDH (U/L)	876 / 1,096 / 1,121	858 / 1,092 / 876	619 / 1,066 / 755	714 / 821 / 798
IL-6 (pg/mL)	21.5 / 110.0 / 133.8	27.4 / 18.6 / 17.0	6.4 / 4.8 / 8.1	132.0 / 127.0 / 110.0
PTT (s)	28.4 / 31.0 / 54.1	120.0 / 120.0 / 32.6	31.3 / 35.9 / 22.5	21.0 / 49.0 / 28.6
INR	1.03 / 1.06 / 1.29	1.23 / 1.09 / 1.43	1.06 / 1.03 / 1.12	0.95 / 1.02 / 0.98
Lactate (mmol/L)	21.0 / 17.0 / 8.5	35.0 / 18.9 / 12.0	15.0 / 44.0 / 14.3	10.2 / 9.5 / 13.7
Ferritin (ng/mL)	222.7 / 378.3 / 659.3	312.2 / 210.0 / 288.1	94.4 / 89.6 / 129.0	845.0 / 739.0 / 731.0
ESR (mm/h)	68 / 81 / 66	91 / 92 / 92	81 / 35 / 45	88 / 90 / 91
CRP (mg/L)	3.2 / - / 3.0	84.9 / 112.0 / 114.0	151.6 / 10.5 / 5.8	143.0 / 125.0 / 120.0
Albumin (g/dL)	3.2 / - / 3.0	2.9 / - / 2.8	3.2 / - / 3.0	2.9 / - / 2.6
MAP (mmHg)	73 / 71 / 70	95 / 102 / 102	93 / 73 / 88	77 / 73 / 75
SpO₂ (%)	88 / 87 / 72	81 / 84 / 72	85 / 89 / 96	89 / 91 / 97

In line with the manufacturer’s recommendation for integrated CytoSorb therapy, blood flow rates were set between 200 and 250 mL/min to ensure optimal adsorber performance (7–10). The dialysis dose was maintained at 25–30 mL/kg/h.

The adsorber was replaced after 12 h for the initial session and every 24 h for subsequent sessions. Treatment was discontinued when there were clear signs of improvement in oxygenation (PaO2/FiO2 ratio above 250 mm Hg), reduced vasopressor needs, and/or decreased inflammatory markers, such as serum ferritin.

In one case, CytoSorb treatment was halted after the second session due to severe thrombocytopenia (platelet count <20,000/μL). We assessed laboratory biomarkers related to COVID-19, including procalcitonin (PCT), C-reactive protein (CRP), and ferritin, along with hemodynamic parameters like vasopressor requirements.

We also analyzed lung function (PaO2/FiO2 ratio) and the Sequential Organ Failure Assessment (SOFA) score before and after CytoSorb therapy (11).

## Data collection and analysis

**Figure 1:**
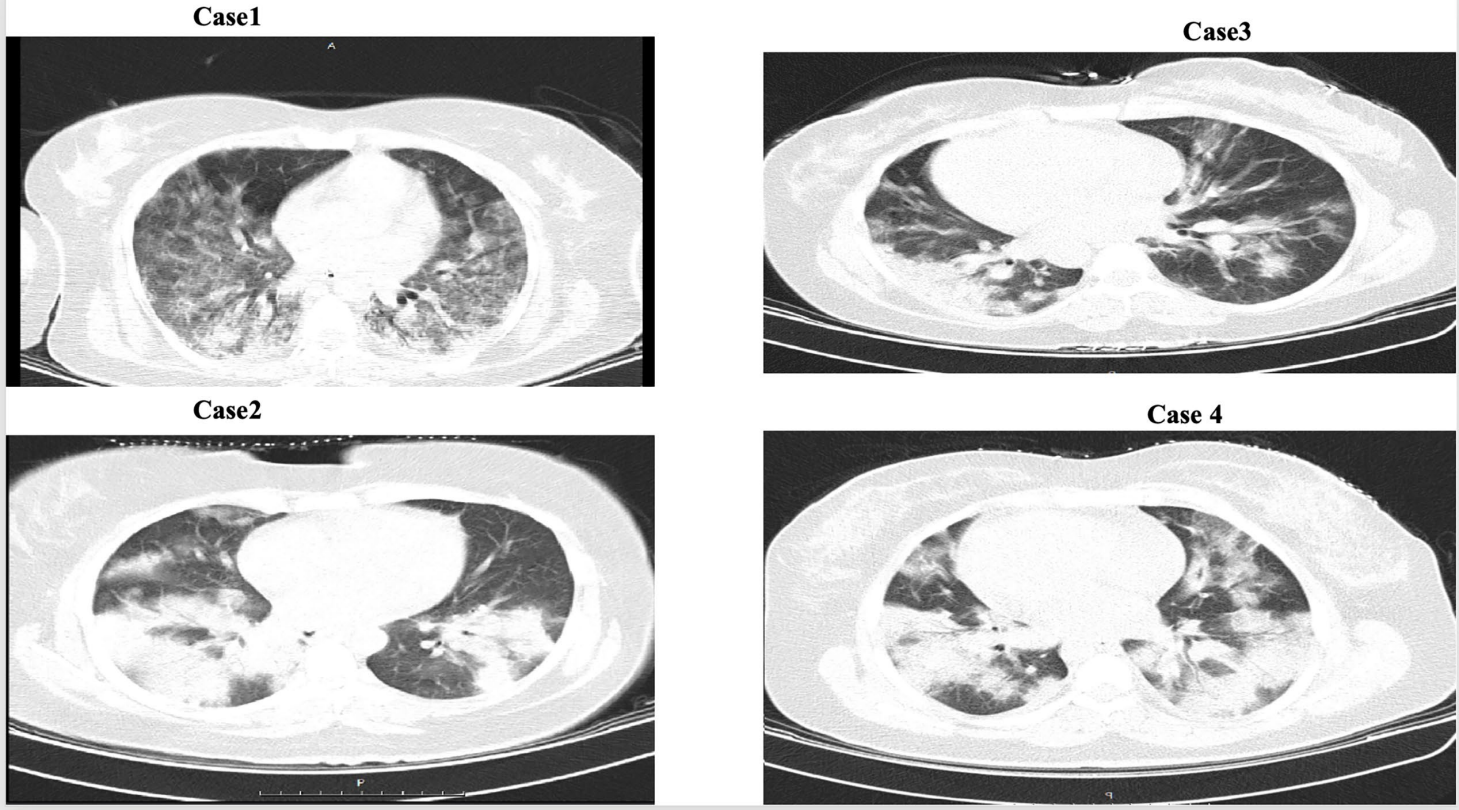
CT scan of the lung patients.

Clinical and laboratory data were collected at three defined time points: immediately before the initiation of the first CytoSorb session (T0), 24 h after initiation (T24), and 48 h after initiation (T48). The parameters analyzed included the Sequential Organ Failure Assessment (SOFA) score for organ function, the Noradrenaline Equivalent Dose (NED) for vasopressor requirements, the PaO₂/FiO₂ ratio for respiratory function, and the serum levels of the inflammatory biomarkers C-reactive protein (CRP) and ferritin (Figure 1).

## Case summaries

### Case 1

A 31-year-old woman, G2 P1 L1, at 23 weeks + 2 days gestation, presented with cough, dyspnea, and chills. Her medical history was notable only for a previous cesarean section and a penicillin allergy. On admission, her temperature was 38.5 °C, MAP was 70 mmHg, and SpO₂ was 88% on room air. She was treated with remdesivir and a methylprednisolone pulse. Anticoagulation was initiated for an acute DVT, and insulin was started for new-onset hyperglycemia. The patient developed AKI, and CVVH with integrated CytoSorb therapy was initiated on the first day of ICU admission. Mechanical ventilation was not required. Her condition improved steadily, and she was discharged in stable condition on prophylactic low molecular weight heparin. At follow-up, there were no complications during delivery or for the newborn.

### Case 2

A 32-year-old woman, G5 P3 L1 A1, at 28 weeks + 2 days gestation, was admitted with cough, myalgia, dyspnea, and fatigue. Her medical history was unremarkable. Admission vital signs included a temperature of 38.1 °C, MAP of 95 mmHg, and SpO₂ of 85%. Treatment included prednisolone, remdesivir, and therapeutic anticoagulation for a confirmed DVT. She developed AKI and ARDS on day 8 of her ICU stay, requiring endotracheal intubation and mechanical ventilation for 7 days. CVVH with integrated CytoSorb was initiated concurrently for 42 h. She made a full recovery and was discharged on subcutaneous low molecular-weight heparin.

### Case 3

A 29-year-old woman, G4 P1 L1 A2, at 24 weeks + 5 days gestation, presented with cough, dyspnea, headache, chills, and diarrhea. Her medical history included diabetes mellitus and hypothyroidism, managed with metformin and levothyroxine. On admission, her temperature was 38.8 °C, MAP was 90 mmHg, and SpO₂ was 88%. She was treated with remdesivir and dexamethasone. The patient developed ARDS and AKI, requiring mechanical ventilation for 5 days. CVVH with integrated CytoSorb was initiated on ICU day 3 for a total of 155 h. Her condition improved, and she was discharged in stable condition.

### Case 4

A 40-year-old woman, G2 A1, at 34 weeks + 3 days gestation, was admitted with bilateral pneumonia, ARDS, and sepsis, presenting in respiratory distress. Her medical history and admission vital signs were not reported in the available records. She required endotracheal intubation and mechanical ventilation for 4 days. CVVH with integrated CytoSorb was initiated on the first day of ICU admission for 24 h. The CytoSorb treatment was halted after the first session due to severe thrombocytopenia (platelet count <20,000/μL). After her condition improved with continued supportive care, she was discharged from the hospital in a stable condition.

All four patients who received CytoSorb therapy survived and were subsequently discharged from the hospital. The specifics of the CytoSorb treatment and the detailed clinical and laboratory course are summarized in Table 2. Following CytoSorb therapy, we observed a consistent trend of clinical improvement across all patients, as defined by our pre-specified criteria: a reduction in SOFA score, a decrease in vasopressor need (NED), and an improvement in the PaO₂/FiO₂ ratio. The mean SOFA score decreased from 10.5 at T0 to 5.2 at T48. Vasopressor requirements were substantially reduced, with the mean NED decreasing from 0.28 μg/kg/min to 0.05 μg/kg/min over the same period. Lung function improved markedly, with the mean PaO₂/FiO₂ ratio increasing from 152 mmHg at T0 to 245 mmHg at T48 (Table 2).

**Table 2:** Patient-specific CytoSorb treatment parameters and clinical trajectory.

Parameter	Case 1	Case 2	Case 3	Case 4	Mean (T0)	Mean (T48)
Treatment parameters						
Time from ICU Admission to AKI/CRRT (days)	1	8	3	1	-	-
Mode of Use	Integrated CRRT	Integrated CRRT	Integrated CRRT	Integrated CRRT	-	-
Blood Flow Rate (mL/min)	200	250	220	240	-	-
Total Treatment Duration (hours)	72	48	96	24	-	-
Number of CytoSorb Devices (n)	3	2	4	1	-	-
Reason for Discontinuation	Clinical Improvement*	Clinical Improvement*	Clinical Improvement*	Severe Thrombocytopenia	-	-
Clinical & hemodynamic parameters						
SOFA Score (T0)	11	9	12	10	10.5	
SOFA Score (T48)	4	5	6	6		5.2
NED (μg/kg/min) (T0)	0.35	0.22	0.30	0.25	0.28	
NED (μg/kg/min) (T48)	0.02	0.08	0.06	0.04		0.05
PaO₂/FiO₂ (mmHg) (T0)	138	145	165	160	152	
PaO₂/FiO₂ (mmHg) (T48)	255	230	240	255		245
Inflammatory biomarkers						
CRP (mg/dL) (T0)	18.5	22.1	16.0	16.2	18.2	
CRP (mg/dL) (T48)	5.8	9.5	4.2	4.9		6.1
Ferritin (ng/mL) (T0)	1,600	1800	1,100	1,300	1,450	
Ferritin (ng/mL) (T48)	480	700	450	550		545

CRRT, Continuous Renal Replacement Therapy; SOFA, Sequential Organ Failure Assessment; NED, Noradrenaline Equivalent Dose; T0, immediately before first CytoSorb session; T48, 48 h after initiation of CytoSorb therapy.

*Clinical Improvement was defined as a sustained reduction in vasopressor need (NED), improvement in oxygenation (PaO₂/FiO₂ > 250 mmHg), and/or a downward trend in inflammatory markers.

This clinical stabilization was accompanied by a marked and rapid reduction in inflammatory markers. Mean CRP levels fell from 18.2 mg/dL at T0 to 6.1 mg/dL at T48. Similarly, mean ferritin levels dropped from 1,450 ng/mL at T0 to 545 ng/mL at T48.

## Discussion

This case series provides a detailed account of the adjunctive use of CytoSorb hemoadsorption in four critically ill pregnant women with COVID-19, adding to the limited body of knowledge on managing this vulnerable population. The consistent clinical trajectory we observed—characterized by a rapid reduction in vasopressor needs, improvement in oxygenation (PaO₂/FiO₂ ratio), and a concomitant decrease in SOFA scores following CytoSorb initiation—strongly suggests a potential role for cytokine modulation in breaking the cycle of hyperinflammation and organ dysfunction. This aligns with findings in non-pregnant critically ill patients, indicating that extracorporeal hemadsorption therapy can effectively improve hemodynamics and mitigate the inflammatory cascade (12).

The clinical improvement was supported by a marked reduction in inflammatory biomarkers, such as CRP and ferritin, providing a plausible biological rationale for the observed recovery. The management of AKI and the use of extracorporeal blood purification therapies in COVID-19 have been widely debated, with real-world surveys highlighting significant variations in practice and a lack of consensus on their application (1). Within this context, reports on the use of these techniques in pregnant women are exceptionally rare. Our case series therefore contributes a unique perspective, demonstrating the feasible integration of CytoSorb into a CRRT circuit for this specific patient group (13).

Our radiological findings, which showed patchy and ground-glass opacities on chest CT scans, are consistent with previous reports in pregnant patients with COVID-19 pneumonia (14). This underscores the importance of urgent supportive care and the crucial need to distinguish this condition from other pregnancy-related complications like preeclampsia.

Furthermore, the successful outcomes in our series highlight the critical importance of a comprehensive, multidisciplinary approach. The close collaboration between obstetricians, critical care physicians, and other experts was essential in providing integrated care that simultaneously addressed the complexities of maternal critical illness and fetal well-being.

In summary, our findings demonstrate that CytoSorb therapy could be a valuable adjunctive treatment for hyperinflammation in critically ill pregnant COVID-19 patients. However, given the observational nature of this report and the ongoing uncertainties in the field (13), these promising results warrant further investigation. Future research should focus on larger, controlled studies to definitively evaluate the safety, efficacy, and optimal timing of CytoSorb therapy within the broader supportive care spectrum for pregnant women with severe COVID-19, with a continued emphasis on prioritizing both maternal and fetal health.

## Data Availability

The raw data supporting the conclusions of this article will be made available by the authors, without undue reservation.
